# *FLT3*-ITD Allelic Burden and Acute Promyelocytic Leukemia Risk Stratification

**DOI:** 10.3390/biology10030243

**Published:** 2021-03-21

**Authors:** Andrew Y. Li, Sarah M. Kashanian, Bryan C. Hambley, Kyle Zacholski, Vu H. Duong, Firas El Chaer, Noa G. Holtzman, Ivana Gojo, Jonathan A. Webster, Kelly J. Norsworthy, Bruce Douglas Smith, Amy E. DeZern, Mark J. Levis, Maria R. Baer, Farin Kamangar, Gabriel Ghiaur, Ashkan Emadi

**Affiliations:** 1 Department of Medicine, University of Maryland School of Medicine, Baltimore, MD 21201, USA; ali@som.umaryland.edu (A.Y.L.); skashanian@som.umaryland.edu (S.M.K.); vduong@umm.edu (V.H.D.); FE2GH@hscmail.mcc.virginia.edu (F.E.C.); noa.holtzman@nih.gov (N.G.H.); mbaer@umm.edu (M.R.B.); 2 The Sidney Kimmel Comprehensive Cancer Center at Johns Hopkins, Baltimore, MD 21231, USA; bryanhambley@gmail.com (B.C.H.); kyle.zacholski@vcuhealth.org (K.Z.); igojo1@jhmi.edu (I.G.); jwebst17@jhmi.edu (J.A.W.); Kelly.Norsworthy@fda.hhs.gov (K.J.N.); bdsmith@jhmi.edu (B.D.S.); adezern1@jhmi.edu (A.E.D.); levisma@jhmi.edu (M.J.L.); 3 University of Maryland Greenebaum Comprehensive Cancer Center, Baltimore, MD 21201, USA; 4 Department of Biology, School of Computer, Mathematical, and Natural Sciences, Morgan State University, Baltimore, MD 21251, USA; farin.kamangar@morgan.edu; 5 Department of Pharmacology, University of Maryland School of Medicine, Baltimore, MD 21201, USA

**Keywords:** APL, leukemia, *FLT3*-ITD

## Abstract

**Simple Summary:**

Around 12–38% of acute promyelocytic leukemia (APL) patients carry the *FLT3*-ITD mutation, which has been associated with several poor-prognosis indicators such as high white blood cell counts, M3v variant morphology, and the bcr3 isoform. We aimed to retrospectively study the impact of *FLT3*-ITD mutations in APL patients in regard to clinical features, treatment courses, and outcomes. We demonstrate that Sanz high-risk status APL correlates with high *FLT3*-ITD allelic burdens, with every 1% increase in allelic burden correlating with a 0.6 × 10^9^/L increase in white blood cell count (WBC). The presence of *FLT3*-ITD was associated with decreased remission rates and higher 5-year mortality from the time of diagnosis. These findings provide novel revelations regarding the features of *FLT3*-ITD APL, particularly in regard to allelic burden, that warrant further study.

**Abstract:**

The significance of *FLT3*-ITD in acute promyelocytic leukemia (APL) is not well-established. We performed a bi-center retrospective study of 138 APL patients, 59 (42.8%) of whom had *FLT3*-ITD. APL patients with *FLT3*-ITD had higher baseline white blood cell counts (WBCs) (*p* < 0.001), higher hemoglobin, (*p* = 0.03), higher aspartate aminotransferase (*p* = 0.001), lower platelets (*p* = 0.004), lower fibrinogen (*p* = 0.003), and higher incidences of disseminated intravascular coagulation (*p* = 0.005), M3v variant morphology (*p* < 0.001), and the bcr3 isoform (*p* < 0.001). *FLT3*-ITD was associated with inferior post-consolidation complete remission (CR) (*p* = 0.02) and 5-year overall survival (OS) of 79.7%, compared to 94.4% for *FLT3*-WT (wild-type) (*p* = 0.02). *FLT3*-ITD was strongly associated with baseline WBCs ≥ 25 × 10^9^/L (odds ratio (OR): 54.4; 95% CI: 10.4–286.1; *p* < 0.001). High *FLT3*-ITD allelic burdens correlated with high-risk (HR) Sanz scores and high WBCs, with every 1% increase in allelic burden corresponding to a 0.6 × 10^9^/L increase in WBC. HR APL was associated with a 38.5% increase in allelic burden compared with low-risk (LR) APL (95% CI: 19.8–57.2; *p* < 0.001). Our results provide additional evidence that *FLT3*-ITD APL is a distinct subtype of APL that warrants further study to delineate potential differences in therapeutic approach.

## 1. Introduction

First described by Norwegian hematologist Leif Hillestad in 1957 through a series of three cases, acute promyelocytic leukemia (APL) was aptly named for its predominance of promyelocytes and deadly coagulopathy characterized by “a very rapid fatal course of only a few weeks’ duration” [1]. Today with the advent of all-*trans* retinoic acid (ATRA) and arsenic trioxide (ATO), APL is highly curable if treatment is initiated promptly. APL is classified as the French–American–British (FAB) subtype M3 of acute myeloid leukemia (AML), classically arising from a balanced reciprocal translocation between chromosomes 15 and 17. The fusion between the promyelocytic leukemia (PML) and retinoic acid receptor alpha (RARα) genes results in the PML–RARα rearrangement t(15;17)(q24;q21), leading to the disruption of the RARα-regulated maturation of myeloid progenitors at the promyelocytic stage [2]. APL comprises less than 10% of AML, with an estimated 0.01/100,000 incidence in Western countries, affecting men and women equally [3].

FMS-like tyrosine kinase 3 (*FLT3*) is a proto-oncogene implicated in leukemogenesis. The *FLT3* ligand binds to the extracellular *FLT3* receptor, inducing homodimerization that potentiates a downstream signaling cascade involved in the regulation of the proliferation, differentiation, and apoptosis of early myeloid and lymphoid progenitor cells. The most common *FLT3* aberration is a 3-to->400-base-pair in-frame internal tandem duplication (ITD) mutation within exon 14 that leads to the constitutive activation of the *FLT3* receptor and ligand-independent autophosphorylation [4,5,6,7,8,9]. *FLT3*-ITD mutations are found in 20–30% of young adults with AML and are a poor prognostic indicator [9]. Targeted *FLT3* inhibitors have been developed such as giltertinib, which was associated with significantly longer overall survival (OS) and higher complete remission (CR) rates compared to those with salvage chemotherapy in relapsed or refractory *FLT3*-ITD AML [10].

Whereas the importance of *FLT3*-ITD in AML from prognosis to treatment is well known, its significance in APL is less established. Present in 12–38% of acute promyelocytic leukemia [5,6], *FLT3*-ITD has been associated with high white blood cell counts (WBCs), the short PML-RARα breakpoint cluster region 3 (bcr3) isoform, and microgranular variant M3 (M3v) APL [6,7,8,9,11]. The Sanz grouping classifies APL patients as high risk (HR) (WBC > 10 × 10^9^/L), intermediate risk (IR) (WBC ≤ 10 × 10^9^/L, platelets ≤ 40 × 10^9^/L), and low risk (LR) (WBC ≤ 10 × 10^9^/L, platelets > 40 × 10^9^/L) for relapse-free survival (RFS) [12]. Sanz HR APL is associated with *FLT3*-ITD [8,12]. The clinical outcomes in *FLT3*-ITD APL remain controversial, and little is known about the impact of ITD insertion length and allelic burden in APL. In this retrospective study, we investigated the significance of *FLT3*-ITD, insertion length, and allelic burden in APL.

## 2. Materials and Methods

Patients 18 years or older treated at the University of Maryland Medical Center and Johns Hopkins Hospital from January 2000 to May 2020 were included. APL was defined by the cytogenetic and/or molecular confirmation of PML-RARα. *FLT3*-ITD mutations were targeted at the juxtamembrane region of the *FLT3* gene (exons 14–15) via primers, amplified, and identified by fluorescent PCR. The *FLT3*-ITD allelic burden was estimated as a percent ratio of the area under the curve (AUC) of the variant peak divided by the AUC of the wild-type (WT) peak. The ITD insertion length was determined by subtracting the 328-base-pair PCR product of the *FLT3* gene from the base pair size of the variant peak. De-identified patient ages, genders, ethnicities, body mass indices (BMIs), laboratory measurements, Sanz and *FLT3*-ITD statuses, ITD allelic burdens and insertion lengths, induction chemotherapy regimens, and outcomes were inputted into Microsoft Excel. These data were then transferred to Stata, Version 16.1 (StataCorp, College Station, TX, USA). All the statistical analyses were performed and graphs were made using Stata.

The Chi-square test of independence was run for two categorical variables (e.g., Sanz and *FLT3*-ITD status). Mean differences between two groups (e.g., *FLT3*-ITD status) were tested using independent-sample t-tests, and mean differences between three or more groups (e.g., Sanz) were tested using one-way analysis of variance (ANOVA), followed by Scheffe tests. Pearson’s and Spearman’s correlations were used to study the associations of two continuous variables (e.g., allelic burden and WBC). For multiple regression analyses, logistic regression models were used for the associations of independent binary outcomes (e.g., *FLT3*-ITD status) with independent variables of interest (e.g., Sanz), before and after adjustment for other covariates (e.g., age, gender, and ethnicity). Similarly, linear regression models were used for the association of continuous outcomes (e.g., allelic burden) with independent exposures of interest (e.g., Sanz) before and after adjustment for other covariates. Overall survival (OS) was compared between all groups (*FLT3*-ITD, *FLT3*-WT, and Sanz HR/IR/LR) using the log-rank test for the equality of survivor functions and graphed using the Kaplan–Meier method. Cox regression models were used to compare survival between groups after adjustment for covariates of interest.

## 3. Results

We identified 138 patients (47 (34.1%) HR, 54 (39.1%) IR, and 37 (26.8%) LR) (Table 1). *FLT3*-ITD was detected in 59/138 (42.8%) of the APL patients. There were no significant differences in demographics between any of the groups (age, gender, ethnicity, and BMI). The *FLT3*-ITD patients were more likely to possess higher WBC (*p* < 0.001), M3v (*p* < 0.001), and bcr3 (*p* < 0.001) characteristics, reported to be associated with worse outcomes in APL [6,7,8,9,11]. Higher hemoglobin (*p* = 0.03) and aspartate aminotransferase (AST) (*p* = 0.001), lower platelets (*p* = 0.004) and fibrinogen (*p* = 0.003), and a higher incidence of disseminated intravascular coagulation (DIC) (*p* = 0.005) were also noted in our *FLT3*-ITD cohort, albeit without differences in all-cause bleeding (*p* = 0.39), intracranial hemorrhage (*p* = 0.80), or thrombosis (*p* = 0.33). There were no significant differences noted in the incidences of differentiation syndrome when the cohort was stratified by the presence of *FLT3*-ITD (*p* = 0.29), Sanz risk status (*p* = 0.20), and both *FLT3*-ITD and Sanz risk statuses (*p* = 0.22).

Sanz HR patients were more likely to have *FLT3*-ITD compared to IR and LR patients (Chi-square test of independence, *X^2^* (2, N = 138) = 37.8, *p* < 0.001). However, the association between HR status and *FLT3*-ITD (odds ratio (OR): 13.4; 95% CI: 4.7–38.3; *p* < 0.001) disappeared when adjusting for WBCs and platelets (OR: 0.7; 95% CI: 0.1–5.5; *p* = 0.80). Sanz LR and IR patients with WBCs < 10 × 10^9^/L comprised roughly 66% of the cohort, leaving the remaining 34% of HR patients divided evenly into two groups: WBCs of 10–25 × 10^9^/L and >25 × 10^9^/L. *FLT3*-ITD was strongly associated with WBCs ≥25 × 10^9^/L (OR: 54.4; 95% CI: 10.4–286.1; *p* < 0.001), similar to the previously reported WBCs ≥20 × 10^9^/L [7]. WBCs of 10–25 × 10^9^/L were, to a lesser degree, also associated with *FLT3*-ITD (OR: 8.65; 95% CI: 2.71–27.5; *p* < 0.001). No differences in post-induction complete remission (CR) (*p* = 0.42), post-consolidation CR (*p* = 0.61), induction deaths (*p* = 0.80), and OS (*p* = 0.33) were noted between the Sanz risk groups.

HR APL was associated with an ITD insertion length decrease of 20 base pairs compared to LR APL (95% CI: −40.0 to −0.23; *p* = 0.05); however, this finding was lost when adjusting for platelets (−13.5; 95% CI: −38.2–11.2; *p* = 0.3). Whereas the ITD insertion length was associated with higher platelet counts (rs = 0.39, *p* = 0.003), no association was noted between the insertion length and WBC (rs = −0.06, *p* = 0.65). A longer ITD insertion length and ITD mutant/wildtype ratio greater than 0.5–0.66 have been associated with shorter RFS, and OS in APL [9,10,11,12,13,14]. No correlation between the insertion length and OS was noted in this study (*p* = 0.38). 

**Figure 1:**
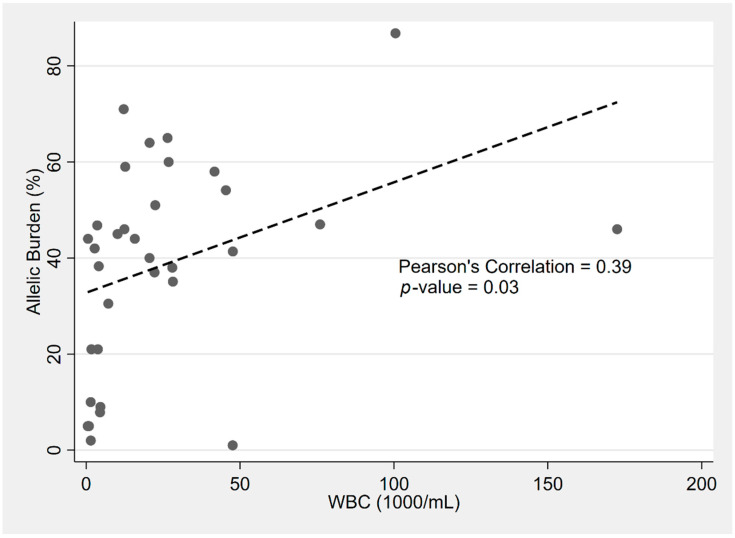
Distribution of *FLT3*-ITD allelic burden (%) and associated WBC count (×10^9^/L) at diagnosis per patient in *FLT3*-ITD APL.

The Sanz risk status significantly correlated with allelic burden according to one-way ANOVA (F(2, 30) = 12.1, *p* < 0.0001). According to post hoc Scheffe tests, the allelic burden differed between HR/IR (*p* = 0.007) and HR/LR (*p* = 0.001), but not in IR/LR (*p* = 0.32). These findings persisted when adjusting for center, age, gender, ethnicity, WBC, and platelets via linear regression. HR APL was associated with a 38.5% increased allelic burden compared with LR APL (95% CI: 19.8–57.2; *p* < 0.001). According to Spearman’s Rho, the *FLT3*-ITD allelic burden was associated with higher WBCs (rs = 0.49, *p* = 0.03). For every 1% increase in allelic burden, the WBC increased by 0.6 × 10^9^/L. The relationship between the *FLT3*-ITD allelic burden and WBC can be visualized in Figure 1. Of note, the majority of the reported allelic ratios (33/59 patients) were from those seen after 2012. There was no significant relationship between the *FLT3*-ITD allelic burden and OS (*p* = 0.97).

**Figure 2:**
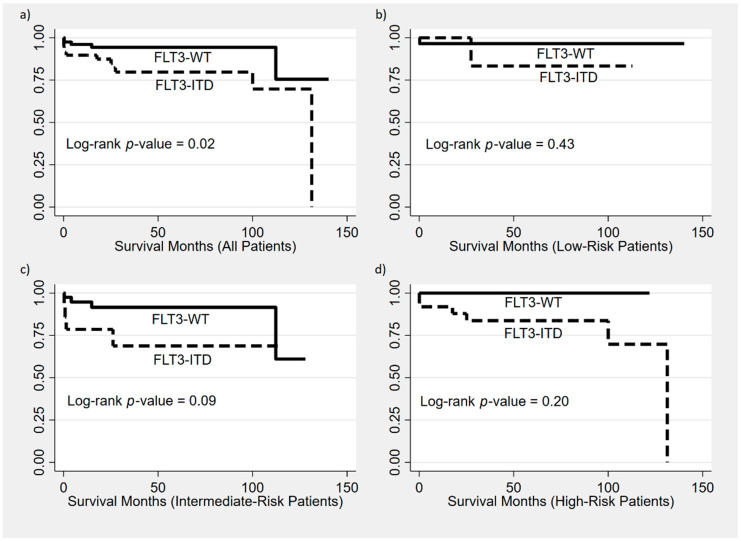
Kaplan–Meier survival curves comparing OS between (**a**) all patients: *FLT3*-ITD vs. *FLT3*-WT, (**b**) *FLT3*-ITD/Sanz LR vs. *FLT3*-WT/Sanz LR, (**c**) *FLT3*-ITD/Sanz IR vs. *FLT3*-WT/Sanz IR, and (**d**) *FLT3*-ITD/Sanz HR vs. *FLT3*-WT/Sanz HR.

While studies have reported a higher incidence of induction death and inferior CR rates, OS, and RFS in *FLT3*-ITD APL [6,9,10,11,15,16], we report no significant differences in the CR duration after induction (*p* = 0.70) or death during induction (*p* = 0.13). We found shorter post-consolidation CR durations (*p* = 0.02) and OS (*p* = 0.02) in *FLT3*-ITD APL (Figure 2). The 5-year OS for patients with *FLT3*-ITD was 79.7% compared to 94.4% for *FLT3* wild-type patients. *FLT3*-ITD was associated with a higher mortality risk, with a hazard ratioof 3.25 (95% CI: 1.14–9.25; *p* = 0.027). Other studies reported no association between *FLT3*-ITD and CR/RFS/OS/early death in APL [7,8,11,17,18,19,20]. The variability of the outcomes may be attributed to the small cohort size, differences in inclusion criteria, varying treatment protocols, and adherence to follow-up.

Pre-arsenic trioxide (ATO) studies showed reduced OS and increased relapse rates with *FLT3*-ITD [9,15,21] in APL, whereas post-ATO studies demonstrated no prognostic significance of *FLT3*-ITD [17,18,19,20], suggesting the effect of *FLT3*-ITD may be mitigated with ATO. Our cohort consisted of 26 *FLT3*-ITD patients treated with ATO, compared to 32 without ATO, and 41 *FLT3*-WT patients treated with ATO, compared to 37 without (two patients with unclear induction regimens). When mutually adjusting for *FLT3*-ITD status, the ATO-containing regimens were not associated with improved OS, with a hazard ratio (95% CI) of 1.13 (0.41–3.16). When stratified by the ATO versus non-ATO regimens, *FLT3*-ITD was associated with increased mortality in both groups. The hazard ratio (95% CI) was 4.78 (1.01–22.6) for the non-ATO regimens and 3.17 (0.58–17.3) for the ATO-containing regimens (*p*-value for interaction = 0.46), suggesting no evidence for the impact of ATO-containing regimens on the prognostic significance of *FLT3*-ITD in this study. However, the study sample size was modest, making the power relatively low for detecting effect modification. The number of patients treated with gemtuzumab ozogamicin was too small (*n* = 4) to perform statistical analysis on outcomes.

## 4. Discussion

APL therapy differs in Sanz HR versus IR/LR groups, and risk-stratifying APL is important for optimizing outcomes. Genes impacting differentiation are downregulated and genes involved in cellular adhesion, invasiveness, and metastasis are upregulated in *FLT3*-ITD APL [16]. Given the associations of *FLT3*-ITD with poor prognostic features and outcomes, *FLT3*-ITD APL has been proposed to be a distinct subtype of APL [7,9]. The high WBCs, M3v, and bcr3 reported in this study have been well-established in *FLT3*-ITD APL, whereas the shorter post-consolidation CR duration and OS found here are less well-established.

We found a strong correlation between *FLT3*-ITD and leukocytosis in APL, suggesting a WBC cutoff of ≥25 × 10^9^/L as an indicator for considering testing for *FLT3*-ITD in APL. The well-known association between leukocytosis and *FLT3*-ITD in APL is further expanded upon in this study by the novel revelation of the WBC’s relationship with the ITD allelic burden, with every 1% increase in allelic burden equating to a 0.6 × 10^9^/L increase in WBC. Interestingly, the allelic burden was not found to be associated with OS. 

## 5. Conclusions

Taken together, these results support the importance of additional study of the significance of the *FLT3*-ITD mutation and ITD allelic burden in APL. More data are required to determine the utility of incorporating *FLT3*-ITD into risk-adapted treatment algorithms and molecular monitoring. The absence of routine testing for *FLT3*-ITD in APL, the lack of international standardized *FLT3*-ITD assays, and the rarity of the disease pose limitations for studies of *FLT3*-ITD APL [6,13].

## Figures and Tables

**Table 1:** Baseline demographic and clinical data of 138-patient acute promyelocytic leukemia (APL) cohort differentiated by presence of *FLT3*-ITD and Sanz risk status.

Variable	ITD (*n* = 59)	WT (*n* = 79)	*p*-Value	HR (*n* = 47)	IR (*n* = 54)	LR (*n* = 37)	*p*-Value	ITD, HR (*n* = 37)	WT, HR (*n* = 10)	ITD, IR (*n* = 14)	WT, IR (*n* = 40)	ITD, LR (*n* = 8)	WT, LR (*n* = 29)	*p*-Value
Age (Years)	47	50	0.27	45	49	53	0.07	45	46	52	48	49	54	0.26
Gender	M (44%) F (56%)	M (52%) F (48%)	0.39	M (49%) F (51%)	M (48%) F (52%)	M (49%) F (51%)	1.0	M (49%) F (51%)	M (50%) F (50%)	M (43%) F (57%)	M (50%) F (50%)	M (25%) F (75%)	M (55%) F (45%)	0.80
Ethnicity	W (53%) B (30%) O (17%)	W (56%) B (20%) O (24%)	0.33	W (44% B (28%) O (28%)	W (65%) B (22%) O (13%)	W (52%) B (24%) O (24%)	0.26	W (46%) B (32%) O (22%)	W (40%) B (10%) O (50%)	W (72%) B (21%) O (7%)	W (63%) B (22%) O (15%)	W (50%) B (38%) O (12%)	W (52%) B (21%) O (27%)	0.39
BMI	32.3	31.8	0.74	31.7	32.2	31.9	0.97	32.3	29.9	33.6	31.7	30.1	32.4	0.91
WBC	29.9	5.1	<0.001	41.8	2.8	1.5	<0.001	45.9	26.7	4.2	2.3	1.5	1.6	<0.001
Hgb	9.7	8.9	0.03	9.7	8.4	10.0	<0.001	9.8	9.0	9.1	8.2	10.1	9.9	0.003
Plt	28.7	50.3	0.004	26.7	19.6	90.6	<0.001	24.8	33.9	17.8	20.3	66.1	97.3	<0.001
Creat	1.01	0.91	0.28	0.91	0.99	0.94	0.76	0.94	0.79	1.04	0.97	1.25	0.86	0.45
AST	44.5	30.2	0.001	49.3	27.6	32.4	<0.001	49.8	47.6	28.4	27.4	48.6	27.9	<0.001
ALT	44.8	33.7	0.09	50.1	29.2	36.9	0.02	51.2	46.1	30.5	28.8	40.3	36.0	0.16
ALP	83.7	81.4	0.73	86.8	68.8	95.9	0.002	87.8	83.1	68.1	69.0	90.0	97.6	0.02
TBili	1.0	0.9	0.42	1.1	0.9	0.8	0.05	1.0	1.2	1.0	0.8	0.6	0.8	0.11
LDH	968.1	1030.5	0.92	1145.4	1233.7	493.3	0.62	1172.9	1046.4	455.1	1506.3	944.5	368.9	0.86
Fib	168.2	225.0	0.003	160.4	183.4	277.3	<0.001	153.8	184.9	159.9	191.6	249.3	285.0	<0.001
Morph	Classic (59%), Variant (41%)	Classic (94%), Variant (6%)	<0.001	Classic (61%), Variant (39%)	Classic (85%), Variant (15%)	Classic (93%), Variant (7%)	0.007	Classic (52%), Variant (48%)	Classic (100%), Variant (0%)	Classic (67%), Variant (33%)	Classic (92%), Variant (8%)	Classic (80%), Variant (20%)	Classic (96%), Variant (4%)	0.001
BCR	1 (26%), 2 (5%), 3 (53%), 1/2 (0%), 2/3 (16%)	1 (62%), 2 (2%), 3 (22%), 1/2(12%), 2/3 (2%)	<0.001	1 (29%), 2 (3%), 3 (52%), 1/2 (3%), 2/3 (13%)	1 (59%), 2 (3%), 3 (22%), 1/2(10%), 2/3 (6%)	1 (50%), 2 (5%), 3 (35%), 1/2 (5%), 2/3 (5%)	0.22	1 (17%), 2 (4%), 3 (62%), 1/2 (0%), 2/3 (17%)	1 (72%), 2 (0%), 3 (14%), 1/2(14%), 2/3 (0%)	1 (33%), 2 (0%), 3 (45%), 1/2 (0%), 2/3 (22%)	1 (70%), 2 (0%), 3 (13%), 1/2(13%), 2/3 (0%)	1 (60%), 2 (20%), 3 (20%), 1/2 (0%), 2/3 (0%)	1 (46%), 2 (0%), 3 (40%), 1/2 (7%), 2/3 (7%)	0.002
DS	Yes (46%), No (54%)	Yes (35%), No (65%)	0.29	Yes (49%), No (51%)	Yes (39%), No (61%)	Yes (30%), No (70%)	0.20	Yes (49%), No (51%)	Yes (50%), No (50%)	Yes (57%), No (43%)	Yes (32%), No (68%)	Yes (12%), No (88%)	Yes (34%), No (66%)	0.22
DIC	Yes (73%), No (27%)	Yes (48%), No (52%)	0.005	Yes (79%), No (21%)	Yes (61%), No (39%)	Yes (30%), No (70%)	<0.001	Yes (81%), No (19%)	Yes (70%), No (30%)	Yes (79%), No (21%)	Yes (55%), No (45%)	Yes (25%), No (75%)	Yes (31%), No (69%)	<0.001
Bleeding	Yes (49%), No (51%)	Yes (41%), No (59%)	0.39	Yes (55%), No (45%)	Yes (48%), No (52%)	Yes (24%), No (76%)	0.01	Yes (57%), No (43%)	Yes (50%), No (50%)	Yes (43%), No (57%)	Yes (50%), No (50%)	Yes (25%), No (75%)	Yes (24%), No (76%)	0.11
ICH	Yes (14%), No (84%)	Yes (11%), No (89%)	0.80	Yes (19%), No (81%)	Yes (13%), No (87%)	Yes (3%), No (97%)	0.06	Yes (16%), No (84%)	Yes (30%), No (70%)	Yes (14%), No (86%)	Yes (12%), No (88%)	Yes (0%), No (100%)	Yes (3%), No (97%)	0.23
Clot	Yes (19%), No (81%)	Yes (11%), No (89%)	0.33	Yes (21%), No (79%)	Yes (7%), No (93%)	Yes (16%), No (84%)	0.12	Yes (22%), No (78%)	Yes (20%), No (80%)	Yes (14%), No (86%)	Yes (5%), No (95%)	Yes (12%), No (88%)	Yes (17%), No (83%)	0.31
CR_induc_	Yes (89%), No (4%), Death (7%)	Yes (93%), No (3%), Death (4%)	0.70	Yes (90%), No (5%), Death (5%)	Yes (93%), No (0%), Death (7%)	Yes (91%), No (6%), Death (3%)	0.42	Yes (88%), No (6%), Death (6%)	Yes (100%), No (0%), Death (0%)	Yes (85%), No (0%), Death (15%)	Yes (95%), No (0%), Death (5%)	Yes (100%), No (0%), Death (0%)	Yes (88%), No (8%), Death (4%)	0.68
CR_cons_	Yes (81%), No (4%), Death (15%)	Yes (97%), No (0), Death (3%)	0.02	Yes (86%), No (6%), Death (8%)	Yes (90%), No (0%), Death (10%)	Yes (93%), No (0%), Death (7%)	0.61	Yes (83%), No (7%), Death (10%)	Yes (100%), No (0%), Death (0%)	Yes (75%), No (0%), Death (25%)	Yes (97%), No (0%), Death (3%)	Yes (86%), No (0%), Death (14%)	Yes (95%), No (0%), Death (5%)	0.26
5-Year OS	79.7%	94.4%	0.02	87.1%	85.5%	92.4%	0.33	83.7%	100%	68.8%	91.6%	83.3%	96.6%	0.13

ITD = *FLT3* internal tandem duplication mutation, WT = *FLT3* wild-type, HR = Sanz high risk, IR = Sanz intermediate risk, LR = Sanz low risk, Age = mean age in years, Gender = male (M) or female (F), Ethnicity (white = W, black = B, other = O), BMI = admission body mass index, WBC = admission white blood cell count in ×10^9^/L, Hgb = admission hemoglobin in g/dL, Plt = admission platelet count in ×10^9^/L, Creat = admission creatinine in mg/dL, AST = admission aspartate aminotransferase in units/L, ALT = admission alanine aminotransferase in units/L, ALP = admission alkaline phosphatase in units/L, TBili = admission total bilirubin in mg/dL, LDH = admission lactate dehydrogenase in units/L, Fib = admission fibrinogen in mg/dL, Morph = classic or variant, BCR = breakpoint cluster region, DS = differentiation syndrome during induction, DIC = disseminated intravascular coagulation during induction, Bleeding = any bleeding event during induction, ICH = intracranial hemorrhage during induction, Clot = any thrombotic event during induction, CR_induc._ = complete remission after induction, CR_cons._ = complete remission after consolidation, OS = overall survival (survivor function %) in 5 years.

## Data Availability

The data presented in this study are available on request from the corresponding author. The data are not publicly available due to the protection of the de-identified patient information.

## Abbreviations

ALP alkaline phosphatase
ALT alanine aminotransferase
AML acute myeloid leukemia
ANOVA analysis of variance
APL acute promyelocytic leukemia
AST aspartate aminotransferase
ATO arsenic trioxide
ATRA all-*trans* retinoic acid
AUC area under the curve
bcr3 breakpoint cluster region 3
BMI body mass index
CR complete remission
DIC disseminated intravascular coagulation
DS differentiation syndrome
FAB French–American–British
Fib fibrinogen
*FLT3*-ITD fms-like tyrosine kinase 3 internal tandem duplication
Hgb hemoglobin
HR Sanz high risk
ICH intracranial hemorrhage
IR Sanz intermediate risk
LDH lactate dehydrogenase
LR Sanz low risk
M3v microgranular variant M3
Morph morphology
OS overall survival
PCR polymerase chain reaction
Plt platelet
PML-RARa promyelocytic leukemia retinoic receptor alpha
RFS relapse free survival
TBili total bilirubin
WBC white blood cell
WT wild-type
